# Clinical applications of UTE-T2* in knee MRI

**DOI:** 10.1007/s00256-026-05138-x

**Published:** 2026-01-25

**Authors:** Karen Y. Cheng, Arya Suprana, Yajun Ma, Dina Moazamian, Saeed Jerban, Christine B. Chung

**Affiliations:** 1 Department of Radiology, University of California San Diego, La Jolla, San Diego, CA USA; 2 Research Service, Veterans Affairs San Diego Healthcare System, La Jolla, San Diego, CA USA; 3 Department of Orthopedic Surgery, University of California San Diego, La Jolla, San Diego, CA USA; 4 Department of Radiology, Veterans Affairs San Diego Healthcare System, La Jolla, San Diego, CA USA

**Keywords:** UTE-T2*, MRI, Knee

## Abstract

Many of the tissues of interest in the evaluation of the knee by magnetic resonance imaging (MRI), including subchondral bone, deep calcified layer of cartilage, menisci, tendons, and ligaments, have very short transverse (T2 and T2*) relaxation times related to their intrinsic structure. These tissues appear anechoic on conventional MRI sequences as signal has already decayed to its minimum when image acquisition begins. Only in the setting of significant injury or degeneration is there detectable signal on conventional MRI sequences. Ultrashort echo time (UTE) MRI, which allows for the qualitative and quantitative assessment of short T2 tissues in their normal states, offers a unique opportunity to detect and intervene upon pathological changes early to prevent irreversible damage. Changes on UTE-T2* imaging allow for the identification of subtle alterations in collagen structure, hydration status, and mineralization of tissues that precede morphologic changes visible on conventional imaging. Early detection of such microstructural changes can allow for the earlier diagnosis of tendinopathy, meniscal injury or degeneration, and early osteoarthritis, potentially allowing for improved patient outcomes through earlier intervention. This review will focus specifically on the clinical applications of one UTE MRI technique, UTE-T2*, in the evaluation of musculoskeletal tissues about the knee.

## Introduction

Knee pain is one of the most common musculoskeletal complaints, affecting a wide spectrum of patients from young athletes to older adults with degenerative joint disease. Accurate diagnosis of the underlying cause of knee pain is essential for guiding treatment and preventing chronic disability. While clinical examination and radiography provide useful first-line information, their ability to evaluate soft tissue structures is limited. MRI has therefore become an important tool in the noninvasive assessment of the knee, providing excellent soft tissue contrast and the ability to evaluate cartilage, menisci, ligaments, tendons, and bone marrow in a single examination. Despite these strengths, conventional MRI has intrinsic limitations in imaging many of the tissues of interest in the knee. The menisci, ligaments, deep calcified layer of cartilage, and subchondral bone all contain highly organized intrinsic structures and varying degrees of mineralization, features that result in very short transverse (T2 and T2*) relaxation times. With conventional MRI techniques, which employ echo times (TE) of at least several milliseconds, signal from these short-T2 components has already decayed to zero or near-zero at the time of image acquisition. As a result, these tissues appear uniformly dark on routine sequences, and signal is typically only observed when structural disruption is advanced enough to prolong T2 relaxation times. This limitation makes it challenging to identify early microstructural changes that may precede gross morphologic damage or symptomatic disease.

UTE-MRI addresses this challenge by acquiring signal almost immediately after excitation utilizing TE values that are 100 μs or less, at least 20–50-fold shorter than the shortest conventional MR imaging echo times. This allows for both qualitative and quantitative assessments of tissues with very short transverse relaxation times. By capturing signal before it decays, UTE-MRI allows for visualization of structures that were previously “invisible” on standard MRI. Quantitative UTE techniques further provide opportunities for tissue characterization and biomarker development, holding promise for earlier detection and improved assessment of musculoskeletal disorders such as osteoarthritis and ligament, tendon, or meniscal injury.

The purpose of this review is to summarize the current clinical applications of UTE-T2* MRI in the evaluation of musculoskeletal tissues about the knee. We highlight how UTE-T2* has been used to assess cartilage, subchondral bone, meniscal, ligamentous, and tendinous pathology, emphasizing its role in detecting early microstructural changes and complementing conventional MRI in the assessment of knee disorders.

## UTE imaging of cartilage

### Cartilage degeneration

Articular cartilage has a layered architecture, with variations in collagen organization of each histologic layer accounting for their variable transverse relaxation times. The deeper calcified and radial layers of cartilage, as well as the subchondral bone plate, have very short transverse relaxation times that may be challenging to assess using conventional morphologic or T2 mapping techniques [1, 2]. Although historically chondral abnormalities have been thought to progress from superficial to deep, there has been increasing recognition that these deeper layers are important to the pathogenesis of chondral injury and degeneration [3–6]. Application of UTE MRI techniques, including UTE-T2*, has enabled better identification and both qualitative and quantitative characterizations of these short T2 tissues at the osteochondral junction.

**Fig. 1:**
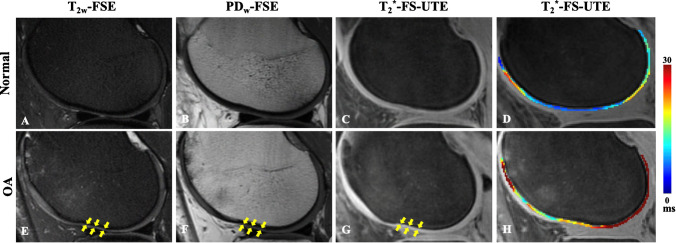
In vivo 3D UTE-T_2_* of femoral OCJ. 2D T_2w_-FSE images (first column; **A**,** E**), PDw-FSE (second column; **B**,** F**), 3D UTE-T_2_* maps (third column; **C**,** G**), and 3D UTE-T_2_* map color overlay (fourth column; **D**,** H**) from a normal knee (top row; **A–D)** and an OA participant (bottom row; **E–F**). The femoral OCJ region is highlighted as a bright signal line in the UTE-T2* image. In the OA knee, the OCJ signals are lost or decreased in the abnormal cartilage regions (arrows). This corresponds to elevated UTE-T_2_* values as demonstrated on the color map

The normal calcified zone of cartilage appears as a linear region of high signal intensity at the subchondral bone interface, with reference T2* values for the calcified layer of cartilage of approximately 1.0–3.3 ms (ms) [7, 8]. In the setting of osteoarthritis, qualitative evaluation of the calcified layer of cartilage may demonstrate regions of discontinuity as well as effacement with ill-definition, the latter corresponding to the histologic finding of duplication of the tidemark [9, 10]. The tidemark is the junction of calcified and uncalcified articular cartilage, and duplication of the tidemark represents reactivation of the calcified layer of cartilage that is seen in osteoarthritis [4, 11]. Quantitative UTE-T2* values at the osteochondral junction have been shown to increase in early osteoarthritis as compared to healthy knees, suggesting that compositional changes in the deep calcified layer of cartilage and subchondral bone may occur before structural changes in proteoglycan and collagen in the superficial cartilage layers detectable by conventional quantitative MRI methods such as T2 or T1ρ (Fig. 1) [12, 13].

**Fig. 2:**
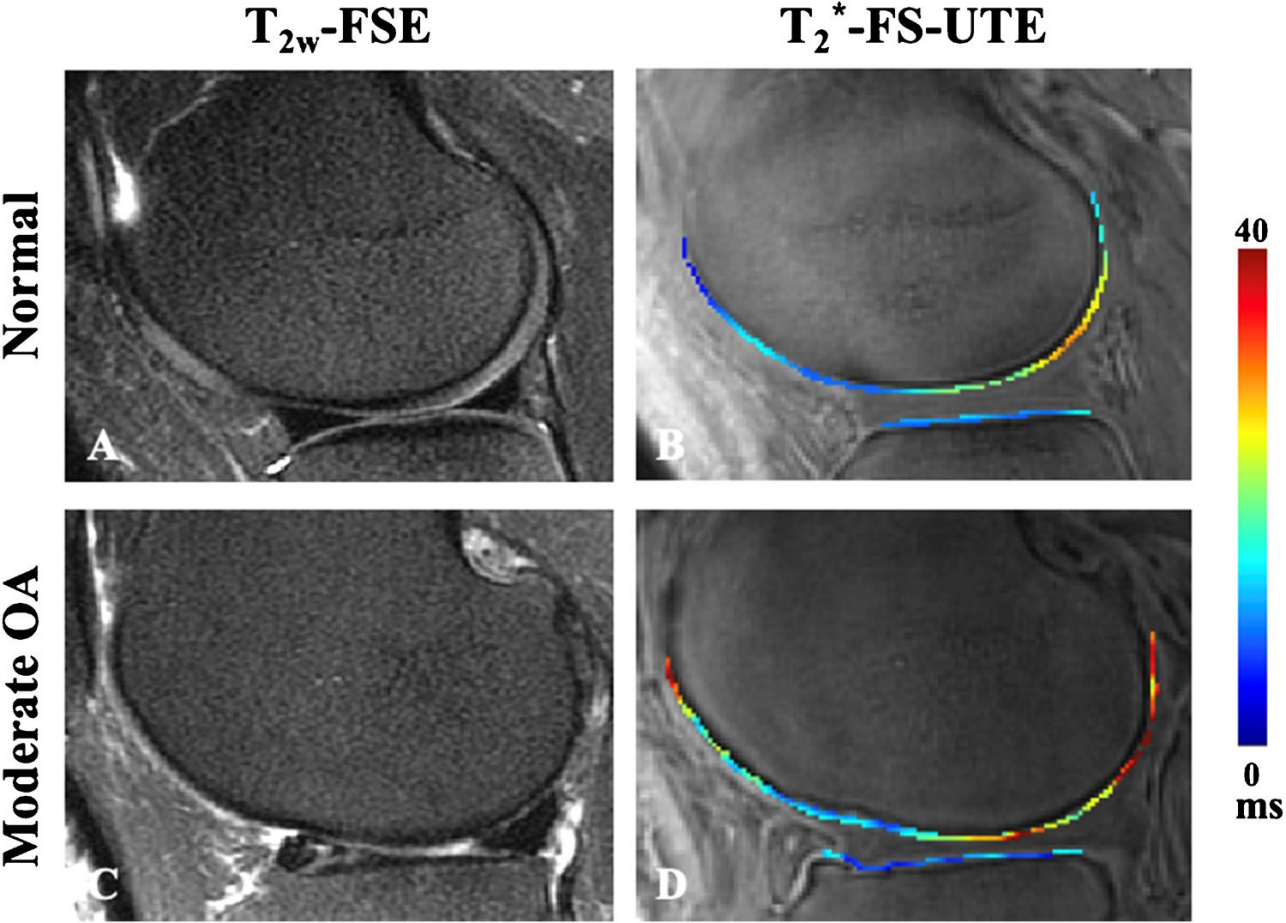
In vivo 3D UTE-T_2_* mapping of cartilage. 2D T_2w_-FSE images (first column; **A**,** C**) and 3D UTE-T_2_* maps (second column; **B**,** D**) were produced from a normal knee and a knee with moderate OA (KL score of 3), respectively. The cartilage UTE-T_2_* values are elevated in the OA knee compared to the normal knee, most apparent along the posterior weight-bearing lateral femoral condyle

When UTE-T2* techniques have been applied to the assessment of chondral degeneration, they have shown significant differences between normal and moderately or severely degenerated cartilage that were not detectable by standard T2 mapping. There have been mixed results regarding whether there are statistically significant differences in early degeneration. For example, in ex vivo studies of anterolateral femoral condyle samples obtained from patients who underwent total knee arthroplasty, UTE-T2* values were significantly different between normal cartilage and moderate or severe degeneration (Fig. 2), but not between normal and mild groups [14, 15]. Similar findings were noted in an ex vivo study of tibial plateau specimens [16]. In contrast, in an in vivo study of patients with knee pain, UTE-T2* values were decreased in participants with mild degeneration as compared to those without chondral degeneration [17]. The authors speculate that this decrease reflects changes in bound water resulting from collagen and proteoglycan degradation but recognize that there may also be a paradoxical increase in T2* values. This is theorized to result from an increase in bound water due to the increased exposed surface area of collagen resulting from collagen fiber destruction with chondral degeneration. The difference in histologic changes between earlier and more advanced stages of chondral degeneration was further explored in a study of cartilage specimens taken from patients who underwent total knee replacement [18]. In this study, T2* was shown to increase in the deep and middle zones of cartilage in early OA, reflecting an increase in water content due to variation in collagen fiber orientation, whereas in advanced OA the T2* was shortened due to a decrease in water content due to proteoglycan loss. Increased sensitivity to structural change in shorter T2 tissues (deep and middle zone cartilage) would be expected with the T2* technique.

### Cartilage injury

In addition to detecting early chondral degeneration, there has been an interest in applying UTE-T2* techniques to evaluate changes in cartilage that occur in the setting of injury and may precede irreversible cartilage damage. Altered mechanical loading and impaction injuries may cause chondrocyte death and tissue damage, both at the articular surface as well as in the deep layer of cartilage and at the osteochondral junction [19]. This has been most well studied in the setting of anterior cruciate ligament (ACL) injury and reconstruction. Despite the intact appearance of the cartilage on both morphologic images and arthroscopy, patients with ACL injuries have been shown to have increased UTE-T2* values at their central medial femoral condylar cartilage as compared to uninjured controls [20]. This is thought to reflect reversible chondral injury that may be treatable with chondroprotective therapies. Subsequent studies have demonstrated that these increased UTE-T2* values persisted for up to 2 years following ACL reconstruction and that higher values were correlated with mechanical factors associated with increased risk and progression of knee osteoarthritis as well as poorer patient-reported outcomes [19, 21–25].

UTE-T2* values in cartilage have also been shown to increase in knee cartilage in the setting of repetitive loading in amateur marathon runners immediately after racing as compared to baseline [26], as well as after mechanical loading exercises [27].

### Cartilage mineralization

Cartilage mineralization typically appears as foci of low signal on the background of higher signal normal hyaline cartilage on conventional MRI. UTE-T2* may, therefore, allow for acquisition of signal from these regions of signal void on routine sequences. One application of this has been in the imaging assessment of hemophilia. In a study of 23 adults with hemophilia, UTE-T2* values for cartilage were shown to be inversely correlated with hemophilia joint health scores [28]. The decrease in UTE-T2* values with progressive joint deterioration was thought to be accounted for by iron deposition within chondrocytes.

Extent of chondral mineralization has also been evaluated utilizing UTE-T2* techniques in the setting of patients with type II diabetes. Compared to non-diabetic controls, subjects with type II diabetes had significantly lower UTE-T2* cartilage values, which reflect increased mineralization of the deep calcified layer of cartilage [29]. Furthermore, independent of diabetic status, UTE-T2* values were negatively correlated with vascular health as assessed by ankle brachial index (ABI), indicating that the extent of chondral mineralization may be correlated with local tissue ischemia and damage.

Mineralization of cartilage and periarticular soft tissues, specifically calcium-containing crystal deposits, has been associated with increased cartilage and meniscus degeneration [30]. However, to our knowledge, UTE-T2* has not yet been applied in the evaluation of chondral chondrocalcinosis, likely because MR imaging has historically had lower specificity and spatial resolution compared to x-ray-based techniques such as CT [31, 32].

## UTE imaging of menisci

As with cartilage, UTE-T2* has also been utilized to assess degeneration, injury, and mineralization of menisci; allowing for assessment of subsurface changes that may not be detectable by either conventional MRI or arthroscopy. Like cartilage, meniscus has a highly organized collagen structure with zonal variation, and it is this specialized structure that enables the meniscus to perform its roles in load-bearing, load transmission, and shock absorption[33]. The peripheral third of the meniscus, or the red zone, is vascularized and has higher T2 values as compared to the inner avascular white zone or the intervening red-white zone[34]. Conventional MRI may fail to detect subtle changes in the meniscus, particularly the white zone, due to its short T2 properties. UTE-T2* imaging, however, allows for direct visualization and quantitative assessment of both the red and white zones. In one in vivo study, the mean UTE-T2* values of the red, red-white, and white zones were 12.6 ms, 6.5 ms, and 7.6 ms, respectively [35]. The same study also showed detectable increases in signal intensity in all zones of the meniscus following contrast administration, which was not possible with conventional MRI techniques [36].

**Fig. 3:**
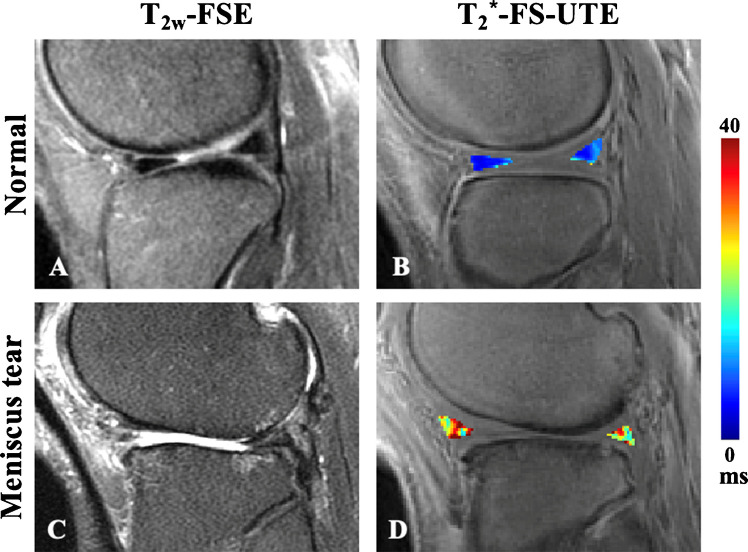
In vivo 3D UTE-T_2_* mapping of meniscus. 2D T_2w_-FSE images (first column; **A**,** C**) and 3D UTE-T_2_* maps (second column; **B**,** D**) were produced from a normal knee and a knee with meniscus tears, respectively. The meniscus UTE-T_2_* values are elevated in the torn meniscus compared to the normal one

UTE-T2* values have been shown to be elevated in the setting of meniscal degeneration as compared to controls, and even higher in the setting of meniscal tear, with reference mean values of 3.6 ms, 7.4 ms, and 9.8 ms for normal, degenerated, and torn menisci, respectively, reported in one study of cadaveric menisci (Fig. 3) [37]. Higher UTE-T2* values have been reported in vivo for normal menisci (8–12 ms) [38, 39]. These changes in UTE-T2* value with degeneration or injury likely reflect histologic changes in collagen architecture, water content, cellularity, and proteoglycan content that may not be detectable with conventional T2 or T2* sequences at early stages [40]. Additionally, UTE-T2* values have been shown to increase in the posterior horn of the medial meniscus in patients with anterior cruciate ligament (ACL) injuries as compared to asymptomatic controls, even in the absence of a visible meniscal tear on conventional MRI or arthroscopy, suggesting that this technique may be applied in detecting subclinical meniscal injuries [39].

UTE-T2* has also been applied in the evaluation of chondrocalcinosis (calcium pyrophosphate dihydrate [CPPD] deposition) in human menisci, with calcifications appearing as dark regions within the less vascular white and red-white zones of the meniscus, with corresponding lower UTE-T2* values in the presence of calcification as compared to healthy controls [41]. This study further demonstrated that menisci with crystal deposition had higher indentation stiffness than normal menisci. This alteration in the mechanical property of the meniscus likely affects meniscal load-bearing and could potentially explain the association of crystal deposition with increased cartilage and meniscal degeneration.

## UTE imaging of knee ligaments

UTE-T2* techniques have also been applied in the evaluation of both native and reconstructed ligaments. Reference UTE-T2* values for the native ACL (11.3–12.3 ms) are slightly higher than those for the PCL (6.9–9.0 ms) [42]. Partial and chronic tears of the native posterior cruciate ligament (PCL) can be challenging to diagnose on MRI but can produce biomechanical alterations that lead to accelerated arthritis[43]. In cadaveric knee specimens, UTE-T2* values have been shown to significantly increase in the setting of both partial and complete PCL transection [44]. The same study showed no detectable change in PCL signal as detected on conventional T2* maps, demonstrating the advantage of UTE imaging in quantitative assessment of the PCL. Quantitative UTE-T2* evaluation of the native PCL may also be valuable in the assessment of knee osteoarthritis, as PCL degeneration has been associated with cartilage loss [45]. UTE-T2* mapping of the PCL in patients with osteoarthritis who subsequently underwent total knee replacement showed higher UTE-T2* values in these patients as compared to asymptomatic volunteers [46]. The UTE-T2* values correlated with the degree of PCL degeneration as characterized by disorganized collagen structure and mucinous degenerative changes on histologic evaluation.

With regards to reconstructed ligaments, ACL grafts are known to undergo a process of revascularization, remodeling, cytologic rearrangement, and adaptation to the new biological and mechanical environment over time called “ligamentization” [47, 48]. While the change in the graft appearance during this time may be minimal on conventional MRI, quantitative assessment with UTE-T2* techniques has shown predictable changes in graft signal. UTE-T2* values increase until approximately 6 months after surgery and then decrease by 12 months [47, 48]. In addition, UTE-T2* has been shown to correlate with the elastic modulus of cadaveric ACL specimens[49]. Higher graft tension, which is a reported risk factor for recurrent ACL injury and residual instability, is also associated with a higher UTE-T2* value [50]. Together, these findings suggest that UTE-T2* techniques may provide a useful non-invasive means of assessing graft maturation and function.

## UTE imaging of tendon

**Fig. 4:**
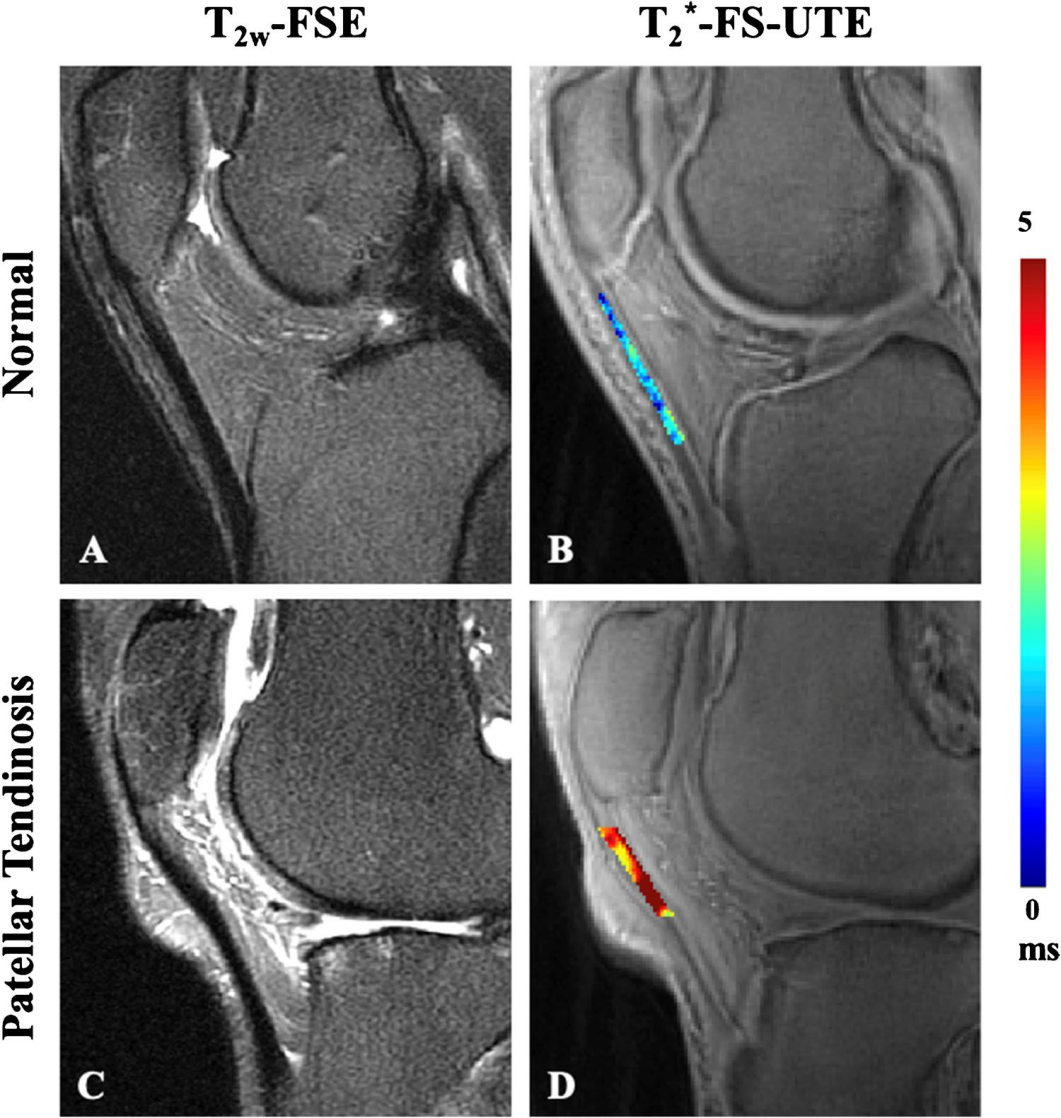
In vivo 3D UTE-T_2_* mapping of tendon. 2D T_2w_-FSE images (first column; **A**,** C**) and 3D UTE-T_2_* maps (second column; **B**,** D**) were produced from a normal knee and a knee with patellar tendinosis, respectively. The patellar tendon UTE-T_2_* values are elevated in patellar tendinosis compared to the normal patellar tendon

Normal tendons also have a well-organized internal structure comprised of collagen fibers and proteoglycans. In tendinopathy (tendinitis, tendinosis), a common tendon disorder, the collagen bundles become disorganized and there is an increase in ground substance and tenocytes with abnormal nuclei [51]. These histopathologic changes are correlated with increased T2* values [52]. At the knee, patellar tendinosis is a condition resulting from overuse that is commonly observed in athletes performing repetitive jumping activities, which results in load-related pain and degenerative tissue changes that occur at the site of the patellar tendon attachment to the patella[53]. The patellar tendon has been evaluated using UTE-T2* imaging [42, 54–56], with reference values on the order of 3–8 ms [42, 52, 57]. In patients with early tendinopathy, the UTE-T2* of the patellar tendon is increased compared to healthy controls (Fig. 4), suggesting that this imaging technique can detect changes in the composition and microstructure of the patellar tendon that occur with degeneration [58]. Furthermore, the increased UTE-T2* values are negatively correlated with the mechanical properties of the tissue as characterized by the tendon modulus[52]. Increased UTE-T2* values in patellar tendinopathy have also been shown to be correlated with patient symptoms, with UTE-T2* values shown to decrease in association with improving clinical outcomes in response to exercise therapy [59].

The quadriceps tendon has also been evaluated with UTE imaging techniques, with one study reporting mean reference UTE-T2* values of approximately 1–12 ms [56, 60]. To our knowledge, UTE-T2* imaging has not yet been applied to the clinical assessment of the native quadriceps tendon, likely due to the lower prevalence of quadriceps tendinopathy (0.2–2% of athletic populations) as compared to patellar tendinopathy (up to 45% of elite jumping athletes)[61]. However, the quadriceps tendon utilized as graft material for anterior cruciate ligament reconstruction has been evaluated using UTE-T2* mapping, with decreasing UTE-T2* values correlating with graft maturation[62].

## Conclusions

UTE-T2* techniques provide a non-invasive means to qualitatively and quantitatively assess multiple structures in the knee, including cartilage, meniscus, ligament, and tendon. It allows for the acquisition of signal from structures that have no detectable signal with conventional sequences, enabling earlier and more sensitive detection of internal derangements of the knee, such as in the setting of early osteoarthritis, subsurface meniscal injury, ligament tears, and patellar tendinopathy. Additionally, in many cases, changes in UTE-T2* values have been shown to correlate with clinical symptoms and functional measures that may normalize with treatment. Together, these observations indicate that UTE-T2* imaging may be applied not only in early injury detection, allowing for intervention to prevent progression to irreversible injury, but also in the monitoring of treatment efficacy of these interventions.

## Data Availability

Not applicable.
